# Study of epinephrine-induced cardiovascular adverse events in patients with anaphylaxis using two spontaneously reported adverse event databases from 2004 to 2024

**DOI:** 10.1038/s41598-025-20999-3

**Published:** 2025-10-23

**Authors:** Priyanka Kamath, Rashmi R. Rao, Ashwin Kamath

**Affiliations:** Department of Pharmacology, Kasturba Medical College Mangalore, Manipal Academy of Higher Education, Manipal, India

**Keywords:** Epinephrine, Anaphylaxis, Stress cardiomyopathy, Adverse drug reaction, Pharmacovigilance, Allergy, Drug safety, Pharmacology

## Abstract

Anaphylaxis is a life-threatening hypersensitivity reaction requiring prompt administration of epinephrine. Despite its life-saving role, hesitancy in epinephrine use persists, largely due to concerns about cardiovascular adverse events (AEs). This study aimed to characterize potentially epinephrine-induced cardiovascular AEs in anaphylaxis using data from two major pharmacovigilance databases. A retrospective analysis was conducted using individual case safety reports (ICSRs) from the US FDA Adverse Event Reporting System (FAERS) and the European EudraVigilance database from 2004 to 2024. ICSRs were included if epinephrine was a suspect drug and the indication involved anaphylaxis or related terms. Reports were screened for cardiovascular AEs and assessed for causality by two independent reviewers. Descriptive statistics were used to summarize findings. A total of 802 and 517 ICSRs were analyzed from FAERS and EudraVigilance, respectively. Cardiovascular AEs were reported in 142 and 134 ICSRs, respectively, with epinephrine as the sole suspect in most cases. Female patients were more frequently represented. Stress cardiomyopathy, myocardial ischemia, tachycardia, and acute myocardial infarction were the most common cardiovascular AEs. Intravenous administration was frequently associated with serious outcomes. Causality assessment indicated that a majority of cardiovascular AEs were likely related to epinephrine. Cardiovascular AEs, particularly stress cardiomyopathy, are associated with epinephrine use in anaphylaxis, especially when administered intravenously. While these findings support cautious use, they also highlight the importance of adhering to recommended intramuscular administration to minimize preventable risks. Clinician awareness and adherence to guidelines remain critical for safe and effective anaphylaxis management, prioritizing cautious use over hesitancy.

**Supplementary Information:**

The online version contains supplementary material available at 10.1038/s41598-025-20999-3.

## Introduction

Anaphylaxis, a type 1 hypersensitivity reaction, can be a life-threatening medical emergency. The lifetime prevalence of anaphylaxis is estimated at 0.05%–2% in the United States and ~ 3% in Europe^1^. Food has been found to be the most common inciting factor, especially in children, with drugs and venom being the other causes^2^. Owing to its lifesaving action, early administration of epinephrine is the mainstay of managing a case of anaphylaxis. Positioning the patient in a recumbent position, with airway management and intravenous fluids, is also important. Antihistaminic agents, glucocorticoids, and beta agonists are add-on agents and cannot substitute for the use of epinephrine^3,4^.

Owing to the suddenness and the extreme emergency nature of anaphylaxis, prospective studies are often not feasible. Various retrospective observational studies have been done to study the management of anaphylaxis. It has been observed that management patterns vary widely from the recommended guidelines^5^. The usage of epinephrine is rare, and antihistaminics are more often used, both during treatment and on discharge^6^. Possible reasons for the lack of adequate use of a guideline-recommended treatment could be an apprehension about adverse events associated with epinephrine such as lactic acidosis, and rare but serious adverse events such as ventricular arrhythmias, ischemic ECG changes, pulmonary edema, though these are known to occur with inadvertent overdose or with intravenous route of epinephrine administration^7,8^.

Adverse event databases that contain spontaneously reported potentially drug-induced untoward events have been widely used to study the safety characteristics of a drug. Despite limitations regarding the data quality and completeness of reporting, the large volumes of adverse events reported over time provide easily-accessible, valuable information on adverse drug reactions, which is difficult to obtain from prospective studies in a controlled environment. In the context of epinephrine use for anaphylaxis, such adverse event databases may provide information regarding the adverse events, which may help to substantiate or refute the hesitancy in the use of epinephrine. In this study, we used two adverse event databases, the United States Food and Drug Administration Adverse Event Reporting System (FAERS) database and the European EudraVigilance, to identify the adverse events that occurred following epinephrine use for anaphylaxis. We further characterized these adverse events into cardiovascular and non-cardiovascular events, the former being the primary proposed reason for hesitancy in epinephrine use, and attempted to assess the causality using the available data in the individual case safety reports (ICSRs). We also describe the clinicodemographic characteristics of the cases.

## Methods

This was a retrospective, database-based study of ICSRs submitted to FAERS and EudraVigilance from the first quarter of 2004, the year FAERS replaced the legacy AERS system, to the last quarter of 2024. The study protocol was approved by the Institutional Ethics Committee, Kasturba Medical College, Mangalore (20 February 2025, IECKMCMLR02/2025/57).

The FAERS data was accessed using the FAERS public dashboard. The European database of suspected adverse drug reactions (ADR) reports was accessed via the EudraVigilance website (https://www.adrreports.eu/en/search_subst.html). The FAERS public dashboard provides a user interface that enables retrieval of ADR reports based on drug name or ADR term; EudraVigilance permits retrieval of ADRs based on drug name only. The drug names searched were epinephrine, epinephrine bitartrate, epinephrine chloride, and epinephrine hydrochloride. These drug names represent all the generic name options available for epinephrine in the searched databases. Combination of epinephrine with other drugs, such as local anesthetics, was not included in the study. ICSRs containing one of the aforementioned drug names as a suspect medication were included in the analysis if one of the following preferred terms, as per Medical Dictionary for Regulatory Activities (MedDRA), was listed in the indication: anaphylactic reaction, anaphylactic shock, anaphylaxis prophylaxis, anaphylaxis treatment, anaphylactoid reaction, anaphylactoid shock, hypersensitivity, allergy, or type I hypersensitivity. ICSRs that contained a blank field for drug indication were excluded. FAERS as well as EudraVigilance use MedDRA terminologies for coding adverse events and drug indications; this ensures consistency in reporting and analysis. Only ICSRs reported by healthcare professionals were considered. Since ADR databases contain ICSRs reported from other countries as well, only reports from the United States were included from FAERS and reports from the European Economic Area from EudraVigilance, to avoid duplication of reports. Data regarding age, sex, suspect drug name, indication for drug use, concomitant medications, adverse reactions, seriousness, outcome, and availability of supporting literature were collected for each ICSR in the two databases.

After filtering the ICSRs, they were categorized as those reporting a cardiovascular adverse event or not; whether the reported event was a cardiovascular adverse event was determined by manual screening of all the ICSRs independently by two investigators; any discrepancy was resolved by consensus. If an ICSR contained multiple adverse events, the presence of at least one cardiovascular adverse event qualified it to be categorized as such. Further, the causality for the ICSRs reporting a cardiovascular event was assessed independently by two investigators, and differences were resolved by consensus. Given the likelihood of limited data being available in the ICSRs, the causality was categorized as follows: related, may be related, may have contributed, and not related. Causality of related was assigned when there was no other reasonable explanation for the adverse event; may be related, when another reasonable explanation was present; for example, patients receiving cardiovascular medications (although cardiovascular disease may not have been mentioned in Indications) or other concomitant drugs which can potentially cause the reported cardiovascular event; may have contributed, when the patient already has the disease which is reported as an adverse event; for example, patient already was on drugs for heart failure or heart failure mentioned in indications and heart failure is also mentioned as an adverse event; not related, when there is a strong reason available to attribute the adverse event to some other cause. Given that multiple sources may report the same adverse event and these would be assigned different identification numbers, thereby precluding identification of duplicates based on IDs, we used the literature sources cited to identify cases reported by more than one source which describes the same case. This deduplication was performed by manual review of the information in the ICSRs as well as reviewing the information available in the cited literature. The deduplication was performed after the identification of ICSRs reporting a cardiovascular adverse event. Whether the cardiovascular event(s) reported in each ICSR was a serious adverse event was evaluated by the investigators based on the adverse event terms reported and the outcome to determine the seriousness of the reaction, irrespective of the seriousness indicated in the ICSR.

The data from the adverse event databases were downloaded and imported into a Microsoft Excel file. Descriptive statistics were used, with the data presented as numbers and percentages.

## Results

From 2004 to 2024, 5019 and 1821 ICSRs were reported by healthcare professionals with epinephrine as a suspect medication in the United States and the European Economic Area, respectively; after filtering the ICSRs based on the indications of interest, 802 and 517 ICSRs were available for analysis, respectively.

The majority of the ICSRs reported epinephrine as the sole suspect drug (Table 1). More than half of the ICSRs reported female sex. Concomitant medications were present in about 30% ICSRs. Around 25% of the ICSRs reported data from published literature.

**Table 1:** Characteristics of the individual case safety reports of epinephrine-induced adverse events in patients with anaphylaxis or related indications.

	FAERS % (*N*)	EudraVigilance % (*N*)
**Number of ICSRs with epinephrine as a suspect drug**	802	517
**Suspect drug**		
Epinephrine only	76.9 (617)	87.2 (451)
Epinephrine + other drug(s)	23.1 (185)	12.8 (66)
**Indications for epinephrine use**		
Anaphylactic reaction	61.2 (491)	34.0 (176)
Anaphylactic shock	2.6 (21)	24.8 (128)
Anaphylaxis prophylaxis	1.4 (11)	6.6 (34)
Anaphylaxis treatment	0.9 (8)	1.2 (6)
Anaphylactoid reaction	0.1 (1)	0.6 (3)
Anaphylactoid shock	0 (0)	0 (0)
Hypersensitivity	28.1 (225)	19.9 (103)
Allergy	10.5 (84)	15.9 (82)
Type I hypersensitivity	0 (0)	0 (0)
**Sex**		
Male	30.8 (247)	38.3 (198)
Female	52.6 (422)	56.3 (291)
Not reported	16.6 (133)	5.4 (28)
**Age**		
2 months-2 years	1.0 (8)	2.5 (13)
3–11 years	5.7 (46)	12.4 (64)
12–17 years	7.9 (63)	9.3 (48)
18–64 years	44.0 (353)	39.8 (206)
65–85 years	6.9 (55)	17.4 (90)
> 85 years	0.2 (2)	0.2 (1)
Not specified	34.3 (275)	18.4 (95)
**Concomitant medication(s)**		
Yes	29.1 (233)	29.4 (152)
No	70.9 (569)	70.6 (365)
**ICSR data mined from literature**	31.2 (250)	18.0 (93)

*FAERS* Food and Drug Administration adverse event reporting system, *ICSR* individual case safety report.

In FAERS and EudraVigilance, 204 and 139 ICSRs reported one or more cardiovascular adverse events, respectively; after deletion of duplicates, 142 and 134 ICSRs were available for analysis, respectively (Table 2). Again, epinephrine was the only suspect drug in the majority of the ICSRs. About 60% of ICSRs reported female sex. Concomitant medications were present in 50% of the reports, and about 50% of the reports were mined from published literature.

**Table 2:** Characteristics of the individual case safety reports of epinephrine-induced cardiovascular adverse events in patients with anaphylaxis or related indications.

	FAERS % (*N*)	EudraVigilance % (*N*)
**Number of ICSRs with epinephrine as a suspect drug**	142	134
**Suspect drug**		
Epinephrine only	71.1 (101)	76.9 (103)
Epinephrine + other drug(s)	28.9 (41)	23.1 (31)
**Indications for epinephrine use**		
Anaphylactic reaction	73.9 (105)	42.5 (57)
Anaphylactic shock	6.3 (9)	29.1 (39)
Anaphylaxis prophylaxis	0 (0)	0 (0)
Anaphylaxis treatment	0.7 (1)	2.2 (3)
Anaphylactoid reaction	0 (0)	1.5 (2)
Anaphylactoid shock	0 (0)	0 (0)
Hypersensitivity	14.8 (21)	15.0 (20)
Allergy	7.7 (11)	13.4 (18)
Type I hypersensitivity	0 (0)	0 (0)
**Sex**		
Male	30.3 (43)	34.3 (46)
Female	58.5 (83)	60.4 (81)
Not reported	11.3 (16)	5.2 (7)
**Age**		
2 months-2 years	0 (0)	1.5 (2)
3–11 years	4.9 (7)	6.7 (9)
12–17 years	5.6 (8)	3.7 (5)
18–64 years	66.2 (94)	60.4 (81)
65–85 years	5.6 (8)	23.1 (31)
Not specified	17.6 (25)	4.5 (6)
**Concomitant medication(s)**		
Yes	45.8 (65)	49.3 (66)
No	54.2 (77)	50.7 (68)
**ICSR data mined from literature**	60.6 (86)	41.0 (55)
**Number of deaths**	4.2 (6)	6.0 (8)

*FAERS* Food and Drug Administration adverse event reporting system, *ICSR* individual case safety report.

On analyzing the ICSRs with cardiovascular adverse events based on causality, the majority of the ICSRs were considered related, with epinephrine being the sole suspect medication in most of them (Table 3). Female sex was more commonly reported. About 25% of the ICSRs with causality ‘related’ reported concomitant medications, and this percentage increased to > 80% for those considered ‘may be related’ or ‘may have contributed’. A larger percentage of reports with causality ‘related’ were judged to be serious. The percentage of reports mined from published literature varied based on the database and causality. Less than 7% of the ICSRs reported death as an outcome, with the highest percentage among ICSRs wherein the causality was judged to be ‘may have contributed’. Data regarding route of drug administration was available only in the EudraVigilance database; of the 134 ICSRs, intravenous route was reported in 45.5% (61) ICSRs, intramuscular in 27.6% (37), subcutaneous in 7.5% (10), and no data was available for 19.4% (26) ICSRs.

**Table 3:** Characteristics of the individual case safety reports of epinephrine-induced cardiovascular adverse events in patients with anaphylaxis or related indications based on causality.

Causality	Related	May be related	May have contributed
**FAERS**			
Total, N	95	18	29
Sex, % (N)			
Male	31.6 (30)	22.2 (4)	31.0 (9)
Female	56.8 (54)	77.8 (14)	51.7 (15)
Not reported	11.6 (11)	0.0 (0)	17.2 (5)
Considered serious by investigators, % (N)	63.2 (60)	66.7 (12)	62.1 (18)
Epinephrine the only suspect drug, % (N)	83.2 (79)	44.4 (8)	48.3 (14)
Concomitant medication, % (N)	27.4 (26)	72.2 (13)	89.7 (26)
Mined from published literature, % (N)	56.8 (54)	77.8 (14)	62.1 (18)
Number of deaths	1.0 (1)	5.6 (1)	13.8 (4)
**EudraVigilance**			
Total, N	87	27	20
Sex, % (N)			
Male	35.6 (31)	29.6 (8)	35.0 (7)
Female	59.8 (52)	59.3 (16)	65.0 (13)
Not reported	4.6 (4)	11.1 (3)	0 (0)
Considered serious by investigators, % (N)	67.8 (59)	81.5 (22)	90.0 (18)
Epinephrine the only suspect drug, % (N)	89.7 (78)	55.6 (15)	50.0 (10)
Concomitant medication, % (N)	26.4 (23)	88.9 (24)	95.0 (19)
Mined from published literature, % (N)	34.5 (30)	59.3 (16)	45.0 (9)
Number of deaths	4.6 (4)	3.7 (1)	15.0 (3)

*FAERS* Food and Drug Administration adverse event reporting system.

A total of 540 and 459 adverse event terms were reported in the ICSRs in FAERS and EudraVigilance, respectively (Supplementary Table 1). Table 4 shows the distribution of the adverse event terms based on causality. Stress cardiomyopathy was the most commonly reported MedDRA preferred term in both databases; tachycardia was the other common preferred term to be reported.

**Table 4:** Common adverse event terms reported in the individual case safety reports of epinephrine-induced cardiovascular adverse events in patients with anaphylaxis or related indications based on causality.

	FAERS		EudraVigilance	
All causality	**Number of AE terms reported**	**540**	**Number of AE terms reported**	**459**
	**Top 5** **AE terms,** % (*N*)			
	Stress Cardiomyopathy	3.1 (17)	Stress cardiomyopathy	5.2 (24)
	Myocardial Ischaemia	2.6 (14)	Tachycardia	4.8 (22)
	Tachycardia	2.2 (12)	Chest pain	2.8 (13)
	Headache	2.0 (11)	Dyspnoea	2.6 (12)
	Acute Myocardial Infarction	1.9 (10)	Headache	2.4 (11)
Related	**Number of AE terms reported**	**353**	**Number of AE terms reported**	**279**
	**Top 5 AE terms**,** % (N)**			
	Stress Cardiomyopathy	4.5 (16)	Tachycardia	6.1 (17)
	Heart Rate Increased	3.1 (11)	Stress cardiomyopathy	5.7 (16)
	Headache	2.5 (9)	Headache	3.6 (10)
	Myocardial Ischaemia	2.5 (9)	Arteriospasm coronary	2.5 (7)
	Acute Myocardial Infarction	2.3 (8)	Chest pain	2.2 (6)
May be related	**Number of AE terms reported**	**69**	**Number of AE terms reported**	**111**
	**Top 5 AE terms**,** % (N)**			
	Chest Discomfort	2.9 (2)	Stress cardiomyopathy	6.3 (7)
	Nausea	2.9 (2)	Dyspnoea	4.5 (5)
	Tachycardia	2.9 (2)	Chest pain	3.6 (4)
	Vomiting	2.9 (2)	Ventricular tachycardia	3.6 (4)
	Acute Coronary Syndrome	1.4 (1)	Myocardial infarction	2.7 (3)
May have contributed	**Number of AE terms reported**	**118**	**Number of AE terms reported**	**69**
	**Top 5 AE terms**,** % (N)**			
	Tachycardia	4.2 (5)	Acute myocardial infarction	5.8 (4)
	Myocardial Ischaemia	3.4 (4)	Myocardial ischaemia	5.8 (4)
	Cardiac Arrest	2.5 (3)	Cardiac arrest	4.3 (3)
	Dyspnoea	1.7 (3)	Chest pain	4.3 (3)
	Hypertension	1.7 (2)	Tachycardia	4.3 (3)

*AE* adverse event, *FAERS* Food and Drug Administration adverse event reporting system.

## Discussion

Between 2004 and 2024, 802 and 517 ICSRs involving epinephrine were analyzed from the United States (FAERS) and European (EudraVigilance) databases, respectively, with most cases identifying epinephrine as the sole suspect drug and a higher reporting among female patients. Cardiovascular adverse events were reported in 204 FAERS and 139 EudraVigilance ICSRs, with epinephrine again being the only suspect in the majority of these cases, and a significant portion of data sourced from published literature. Causality analysis showed that most cardiovascular events were considered “related” to epinephrine, with a higher proportion of serious outcomes and a predominance of female patients. Concomitant medications were present in about 30% of general ICSRs but increased to nearly 50% in cardiovascular-related reports, especially in cases where causality was less certain. The most frequently reported cardiovascular adverse events included stress cardiomyopathy, myocardial ischemia, tachycardia, and acute myocardial infarction, with variations in frequency depending on the assessed causality.

Literature describing prospective studies evaluating the risk of cardiovascular adverse events with epinephrine is not available. However, relevant literature from retrospective studies is available. A study conducted at a Swiss university emergency department to analyze the use of epinephrine in anaphylaxis reported that less than 50% of patients with moderate and severe anaphylaxis were administered epinephrine; the patients were more commonly given either antihistamines or steroids or both, even in the emergency department. The study yielded no information about any cardiovascular adverse events to epinephrine^9^. Another study that assessed the underuse of epinephrine in patients with anaphylaxis in the emergency, found that a higher rate of hospitalization was observed in patients who received epinephrine, the common reasons being acute coronary syndrome, including myocardial infarction. It was also observed that these patients who received epinephrine had higher rates of other inotropic agent usage and fluid challenge^10^. Acute myocardial infarction/myocardial ischemia/arteriospasm coronary were commonly reported ADR terms in our study, particularly within the FAERS dataset; however, the most common cardiovascular MedDRA term altogether was stress cardiomyopathy. A retrospective study which looked at the use of epinephrine for anaphylaxis in older adults (≥ 50 years of age), found that older patients were less likely to receive epinephrine, and among those who did receive epinephrine, they were more likely to have received excessive doses of epinephrine, and cardiac complications to epinephrine were also higher among them. They concluded that intramuscular route of epinephrine was safe in these patients, but intravenous route should be avoided due to the risk of developing cardiac complications^11^. Some of the ADR terms reported in the ICSRs in our study did indicate presence of drug overdose or incorrect route of drug administration. However, manual review of the route of administration showed that epinephrine was administered intravenously in more than half of the ICSRs reporting cardiovascular adverse effects within the Eudravigilance dataset. A systematic review done to review the safety of epinephrine for anaphylaxis has concluded that epinephrine when given through the intramuscular route is safe^12^. There was a prospective study conducted to assess the epinephrine absorption in adults when administered via the subcutaneous versus intramuscular route; the C_max_ was significantly higher when epinephrine was administered via the intramuscular route into the thigh. They observed mild and transient reactions following intramuscular epinephrine, which included pallor, tremors, ‘heart pounding’, dizziness, and headache^13^.

Cardiac adverse effects to epinephrine are known. Activation of the sympathetic system results in a fight-or-flight reaction in the body; in the heart, stimulation of the dominant β1 receptors is responsible for an increase in the heart rate, force of contraction and conduction^14^. Though epinephrine is a life-saving drug in clinical emergencies such as anaphylaxis and cardiac arrest, excessive concentration can increase the myocardial oxygen demand and precipitate angina or myocardial ischemia^15^. Also, it has been observed that epinephrine can reduce the microcirculatory flow in the heart and brain, even after the global blood flow is established, when used for cardiopulmonary resuscitation^15^. Effects of overdose include cardiomyopathy, myocardial ischemia resulting in infarction, pulmonary edema, metabolic acidosis, and potentially fatal arrhythmias. Stress cardiomyopathy, also known as Takotsubo cardiomyopathy, is of particular interest, as it emerged as the most commonly reported cardiovascular adverse event in this study. It involves transient dysfunction of the myocardium, precipitated by physical or emotional stress^16^. Many cases of epinephrine-induced Takotsubo cardiomyopathy have been reported, with events occurring following exposures to low doses of epinephrine in local anesthetic preparation to erroneous intravenous administration of 1:1000 dilution^17,18^. Importantly, full recovery with supportive management is seen in most of the cases. Given that stress cardiomyopathy was noted in both databases, and like an earlier systematic review^19^, more than 50% of the cases occurred following intravenous epinephrine use, it is possible that these incidents may be responsible for the hesitancy noted in the use of epinephrine. However, this also indicates that many of these cases are potentially avoidable by using the right dose and route, and even if stress cardiomyopathy occurs, it is reversible and has a good outcome. The suboptimal use of epinephrine is also acknowledged in the World Allergy Organization anaphylaxis guidance^20^, endorsed by multiple national/other member societies, which recommends early administration of epinephrine intramuscularly into the anterolateral thigh. The current study of two large adverse event databases further supports the recommendations by multiple clinical societies urging the use of intramuscular epinephrine^21–23^.

To the best of our knowledge, this is the first study presenting a comprehensive analysis of potentially epinephrine-induced cardiovascular adverse events in patients with anaphylaxis utilizing spontaneously reported adverse event data in two databases over two decades. The findings support the clinical recommendations to use intramuscular/subcutaneous epinephrine in patients with anaphylactic reaction, while cautioning against inadvertent intravenous injection, which seems to carry a higher risk of serious adverse outcome. Of note, stress cardiomyopathy emerged as the most commonly reported adverse event term in both the studied databases, with a possible female preponderance; these findings need to be considered for addition to the drug label^24,25^. Our study also has certain limitations. The data were obtained from two spontaneously reported adverse event databases which, besides errors, often do not contain all the information required to determine causality. The EudraVigilance access policy limits access to the medical history field and case narrative, which are valuable in determining causality. Therefore, causality as assessed independently by two investigators may not be accurate. Many adverse drug reactions are not reported or underreported. Hence, the actual incidence or prevalence of the adverse events of interest cannot be determined from the databases. Accordingly, the cardiovascular adverse events reported with epinephrine in this study may not necessarily reflect the adverse event patterns in the varied clinical settings.

## Conclusion

This study highlights the occurrence and characteristics of cardiovascular adverse events associated with epinephrine use in anaphylaxis and related conditions, as reported in the FAERS and EudraVigilance databases over a 20-year period. The findings underscore that while epinephrine is a life-saving intervention in anaphylactic emergencies, its administration—especially via the intravenous route—can be associated with serious cardiovascular complications such as stress cardiomyopathy and myocardial ischemia. These events were more frequently reported in female patients. Importantly, inappropriate intravenous administration may contribute to adverse outcomes, many of which are potentially preventable. Given the limitations of spontaneous reporting systems, including underreporting and incomplete data, these findings should be interpreted with caution. Nonetheless, they emphasize the importance of adhering to recommended administration practices and the need for increased awareness and training among healthcare providers to minimize avoidable risks while ensuring timely and effective treatment of anaphylaxis. Overall, while the contention that epinephrine use for anaphylaxis can result in cardiovascular adverse effects is not unfounded, hesitancy overriding cautious administration should be avoided.

## Supplementary Information

Below is the link to the electronic supplementary material.

Supplementary Material 1

## Data Availability

Data is provided within the manuscript or supplementary information files.

## Data availability

Data is provided within the manuscript or supplementary information files.

## References

[1] Yu, J. E. & Lin, R. Y. The epidemiology of anaphylaxis. *Clin. Rev. Allergy Immunol.* **54**, 366–374. 10.1007/s12016-015-8503-x (2018). 26357949 10.1007/s12016-015-8503-x

[2] Lieberman, P. et al. Epidemiology of anaphylaxis: findings of the American college of Allergy, asthma and immunology epidemiology of anaphylaxis working group. *Ann. Allergy Asthma Immunol.* **97**, 596–602. 10.1016/S1081-1206(10)61086-1 (2006). 17165265 10.1016/S1081-1206(10)61086-1

[3] Navalpakam, A., Thanaputkaiporn, N. & Poowuttikul, P. Management of anaphylaxis. *Immunol. Allergy Clin. North. Am.* **42**, 65–76. 10.1016/j.iac.2021.09.005 (2022). 34823751 10.1016/j.iac.2021.09.005

[4] Liberman, D. B. & Teach, S. J. Management of anaphylaxis in children. *Pediatr. Emerg. Care*. **24**, 861–866. 10.1097/PEC.0b013e31818ea116 (2008). quiz 867–9. 19092569 10.1097/PEC.0b013e31818ea116

[5] Banerji, A. et al. Retrospective study of drug-induced anaphylaxis treated in the emergency department or hospital: patient characteristics, management, and 1-year follow-up. *J. Allergy Clin. Immunol. Pract.* **2**, 46–51. 10.1016/j.jaip.2013.08.012 (2014). 24565768 10.1016/j.jaip.2013.08.012

[6] Hitti, E. A., Zaitoun, F., Harmouche, E., Saliba, M. & Mufarrij, A. Acute allergic reactions in the emergency department: characteristics and management practices. *Eur. J. Emerg. Med.* **22**, 253–259. 10.1097/MEJ.0000000000000155 (2015). 24841773 10.1097/MEJ.0000000000000155

[7] Brown, J. C., Simons, E. & Rudders, S. A. Epinephrine in the management of anaphylaxis. *J. Allergy Clin. Immunol. Pract.* **8**, 1186–1195. 10.1016/j.jaip.2019.12.015 (2020). 32276687 10.1016/j.jaip.2019.12.015

[8] Leiser, A., Virdee, R. & Kinniry, P. Severe Epinephrine-Induced Lactic Acidosis. B37. Critical lab value alert; Hormones and electrolytes in the ICU. A3414–A3414 (American Thoracic Society, 2024). 10.1164/ajrccm-conference.2024.209.1_MeetingAbstracts.A3414.

[9] Ehrhard, S. et al. Use of epinephrine in anaphylaxis: a retrospective cohort study at a Swiss university emergency department. *Swiss Med. Wkly.* **153**, 40065. 10.57187/smw.2023.40065 (2023). 36971665 10.57187/smw.2023.40065

[10] Lin, Y-Y. et al. Investigation of the underuse of adrenaline (epinephrine) and prognosis among patients with anaphylaxis at emergency department admission. *Front. Med. (Lausanne)*. **10**, 1163817. 10.3389/fmed.2023.1163817 (2023). 37484849 10.3389/fmed.2023.1163817 PMC10360193

[11] Kawano, T. et al. Epinephrine use in older patients with anaphylaxis: clinical outcomes and cardiovascular complications. *Resuscitation* **112**, 53–58. 10.1016/j.resuscitation.2016.12.020 (2017). 28069483 10.1016/j.resuscitation.2016.12.020

[12] Wood, J. P., Traub, S. J. & Lipinski, C. Safety of epinephrine for anaphylaxis in the emergency setting. *World J. Emerg. Med.* **4**, 245–251. 10.5847/wjem.j.issn.1920-8642.2013.04.001 (2013). 25215127 10.5847/wjem.j.issn.1920-8642.2013.04.001 PMC4129903

[13] Simons, F. E., Gu, X. & Simons, K. J. Epinephrine absorption in adults: intramuscular versus subcutaneous injection. *J. Allergy Clin. Immunol.* **108**, 871–873. 10.1067/mai.2001.119409 (2001). 11692118 10.1067/mai.2001.119409

[14] Motiejunaite, J., Amar, L. & Vidal-Petiot, E. Adrenergic receptors and cardiovascular effects of catecholamines. *Ann. Endocrinol. (Paris)*. **82**, 193–197. 10.1016/j.ando.2020.03.012 (2021). 32473788 10.1016/j.ando.2020.03.012

[15] Gough, C. J. R. & Nolan, J. P. The role of adrenaline in cardiopulmonary resuscitation. *Crit. Care*. **22**, 139. 10.1186/s13054-018-2058-1 (2018). 29843791 10.1186/s13054-018-2058-1 PMC5975505

[16] Bhat, S., Gazi, H., Mwansa, V. & Chhabra, L. Catecholamine-induced reverse Takotsubo cardiomyopathy. *Proc. (Bayl Univ. Med. Cent)*. **32**, 567–569. 10.1080/08998280.2019.1634229 (2019). 31656422 10.1080/08998280.2019.1634229 PMC6793996

[17] Pathangey, G., Moudgal, R., Lee, C. & Henkin, S. Myocardial stunning secondary to erroneous administration of intravenous epinephrine. *SAGE Open. Med. Case Rep.* **11**, 2050313X231159732. 10.1177/2050313X231159732 (2023). 36950049 10.1177/2050313X231159732 PMC10026126

[18] Yamamoto, W. et al. Takotsubo cardiomyopathy induced by very Low-Dose epinephrine contained in local anesthetics: A case report. *Am. J. Case Rep.* **22**, e932028. 10.12659/AJCR.932028 (2021). 34174047 10.12659/AJCR.932028 PMC8244375

[19] Nazir, S. et al. Takotsubo cardiomyopathy associated with epinephrine use: A systematic review and meta-analysis. *Int. J. Cardiol.* **229**, 67–70. 10.1016/j.ijcard.2016.11.266 (2017). 27889211 10.1016/j.ijcard.2016.11.266

[20] Cardona, V. et al. World allergy organization anaphylaxis guidance 2020. *World Allergy Organ. J.* **13** (10), 100472. 10.1016/j.waojou.2020.100472 (2020). 33204386 10.1016/j.waojou.2020.100472 PMC7607509

[21] Whyte, A. F. et al. Emergency treatment of anaphylaxis: concise clinical guidance. *Clin. Med. (Lond)*. **22** (4), 332–339. 10.7861/clinmed.2022-0073 (2022). 35882481 10.7861/clinmed.2022-0073 PMC9345203

[22] ASCIA Guidelines Acute management of anaphylaxis - Australasian Society of Clinical Immunology and Allergy (ASCIA). https://www.allergy.org.au/hp/papers/acute-management-of-anaphylaxis-guidelines. Accessed 10 Aug 2025.

[23] Golden, D. B. K. et al. A 2023 practice parameter update. *Ann. Allergy Asthma Immunol.* **132** (2), 124–176. 10.1016/j.anai.2023.09.015 (2024). 38108678 10.1016/j.anai.2023.09.015

[24] Ramaraj, R. Stress cardiomyopathy: aetiology and management. *Postgrad. Med. J.* **83** (982), 543–546. 10.1136/pgmj.2007.058776 (2007). 17675548 10.1136/pgmj.2007.058776 PMC2600114

[25] Epinephrine Injection USP. U.S. Food and Drug Administration. https://www.accessdata.fda.gov/drugsatfda_docs/label/2021/205029s006lbl.pdf. Accessed 10 Aug 2025.

