# Supplementary material for: Study of epinephrine-induced cardiovascular adverse events in patients with anaphylaxis using two spontaneously reported adverse event databases from 2004 to 2024
Source: Sci Rep. 2025 Oct 23;15:37088. doi: 10.1038/s41598-025-20999-3 (PMC12549901; doi:10.1038/s41598-025-20999-3)
Supplement: Supplementary file 1 — Supplementary Material 1 [file 41598_2025_20999_MOESM1_ESM.docx]

Supplementary Table 1. Adverse event terms reported in the individual case safety reports of epinephrine-induced cardiovascular adverse events in patients with anaphylaxis or related indications based on causality

| **FAERS adverse event terms** | | **EudraVigilance adverse event terms** | |
| --- | --- | --- | --- |
| **All causality** | **N** | **All causality** | **N** |
| Anaphylactic Reaction | 23 | Stress cardiomyopathy | 24 |
| Hypotension | 21 | Tachycardia | 22 |
| Drug Ineffective | 17 | Chest pain | 13 |
| Stress Cardiomyopathy | 17 | Headache | 12 |
| Incorrect Route Of Product Administration | 15 | Anaphylactic reaction | 11 |
| Myocardial Ischaemia | 14 | Dyspnoea | 11 |
| Tachycardia | 12 | Incorrect route of product administration | 11 |
| Headache | 11 | Drug ineffective | 9 |
| Heart Rate Increased | 11 | Myocardial ischaemia | 9 |
| Acute Myocardial Infarction | 10 | Acute myocardial infarction | 8 |
| Cardiac Arrest | 10 | Anaphylactic shock | 8 |
| Dizziness | 9 | Arteriospasm coronary | 8 |
| Hypertension | 9 | Cardiac arrest | 8 |
| Accidental Overdose | 8 | Hypotension | 8 |
| Overdose | 8 | Ventricular tachycardia | 8 |
| Blood Pressure Increased | 7 | Hypertension | 7 |
| Chest Pain | 7 | Palpitations | 7 |
| Kounis Syndrome | 7 | Bradycardia | 6 |
| Nausea | 7 | Incorrect dose administered | 6 |
| Arteriospasm Coronary | 6 | Pallor | 6 |
| Cardiogenic Shock | 6 | Accidental overdose | 5 |
| Cardiomyopathy | 6 | Hyperhidrosis | 5 |
| Loss Of Consciousness | 6 | Kounis syndrome | 5 |
| Accidental Exposure To Product | 5 | Malaise | 5 |
| Chest Discomfort | 5 | Myocardial infarction | 5 |
| Condition Aggravated | 5 | Tremor | 5 |
| Electrocardiogram St Segment Elevation | 5 | Troponin increased | 5 |
| Injection Site Haemorrhage | 5 | Ventricular fibrillation | 5 |
| Off Label Use | 5 | Acute coronary syndrome | 4 |
| Pulmonary Oedema | 5 | Cardiogenic shock | 4 |
| Bradycardia | 4 | Chest discomfort | 4 |
| Dyspnoea | 4 | Electrocardiogram ST segment elevation | 4 |
| Hypoxia | 4 | Heart rate increased | 4 |
| Incorrect Dose Administered | 4 | Accidental exposure to product | 3 |
| Palpitations | 4 | Atrial fibrillation | 3 |
| Abdominal Pain | 3 | Blood pressure increased | 3 |
| Anxiety | 3 | Cardiovascular disorder | 3 |
| Brain Injury | 3 | Device failure | 3 |
| Cardiac Failure Acute | 3 | Drug hypersensitivity | 3 |
| Expired Product Administered | 3 | Ischaemic stroke | 3 |
| Hyperhidrosis | 3 | Medication error | 3 |
| Hypersensitivity | 3 | Nausea | 3 |
| Hypokalaemia | 3 | Sinus tachycardia | 3 |
| Lactic Acidosis | 3 | Vasoconstriction | 3 |
| Myocardial Infarction | 3 | Vomiting | 3 |
| Myocardial Stunning | 3 | Agitation | 2 |
| Pruritus | 3 | Amnesia | 2 |
| Reversible Cerebral Vasoconstriction Syndrome | 3 | Angina pectoris | 2 |
| Toxicity To Various Agents | 3 | Blood pressure decreased | 2 |
| Troponin T Increased | 3 | Cardiac failure | 2 |
| Urticaria | 3 | Cerebral haemorrhage | 2 |
| Ventricular Arrhythmia | 3 | Cold sweat | 2 |
| Ventricular Extrasystoles | 3 | Cyanosis | 2 |
| Vomiting | 3 | Device malfunction | 2 |
| Acute Coronary Syndrome | 2 | Dizziness | 2 |
| Anaphylactic Shock | 2 | Ejection fraction decreased | 2 |
| Atrial Fibrillation | 2 | Electrocardiogram repolarisation abnormality | 2 |
| Cardiac Disorder | 2 | Electrocardiogram ST segment depression | 2 |
| Cerebral Haemorrhage | 2 | Expired product administered | 2 |
| Coronary Artery Thrombosis | 2 | Extrasystoles | 2 |
| Cyanosis | 2 | Injection site pain | 2 |
| Device Failure | 2 | Left ventricular dysfunction | 2 |
| Device Malfunction | 2 | Myocardial oedema | 2 |
| Disseminated Intravascular Coagulation | 2 | Overdose | 2 |
| Drug Interaction | 2 | Peripheral ischaemia | 2 |
| Electrocardiogram Qt Prolonged | 2 | Product administration error | 2 |
| Erythema | 2 | Product dose omission issue | 2 |
| Feeling Abnormal | 2 | Product prescribing error | 2 |
| Flushing | 2 | Product use complaint | 2 |
| Heart Rate Decreased | 2 | Pulmonary oedema | 2 |
| Left Ventricular Dysfunction | 2 | Rash | 2 |
| Long Qt Syndrome | 2 | Sinus bradycardia | 2 |
| Malaise | 2 | Somnolence | 2 |
| Medication Error | 2 | Subarachnoid haemorrhage | 2 |
| Myocardial Injury | 2 | Urticaria | 2 |
| Needle Issue | 2 | Ventricular arrhythmia | 2 |
| Pallor | 2 | Ventricular hypokinesia | 2 |
| Paraesthesia | 2 | Vertigo | 2 |
| Parosmia | 2 | Accidental exposure to product by child | 1 |
| Poor Peripheral Circulation | 2 | Acute respiratory failure | 1 |
| Respiratory Distress | 2 | Altered state of consciousness | 1 |
| Shock | 2 | Arrhythmia induced cardiomyopathy | 1 |
| Troponin Increased | 2 | Arterial occlusive disease | 1 |
| Unresponsive To Stimuli | 2 | Arterial spasm | 1 |
| Vasospasm | 2 | Artery dissection | 1 |
| Ventricular Hypokinesia | 2 | Arthralgia | 1 |
| Ventricular Tachycardia | 2 | Ataxia | 1 |
| Wheezing | 2 | Atrioventricular block | 1 |
| Acquired Left Ventricle Outflow Tract Obstruction | 1 | Autonomic nervous system imbalance | 1 |
| Acute Kidney Injury | 1 | Burning sensation | 1 |
| Acute Pulmonary Oedema | 1 | Cardiac failure congestive | 1 |
| Acute Respiratory Distress Syndrome | 1 | Cardiac fibrillation | 1 |
| Agitation | 1 | Cardiomyopathy | 1 |
| Alopecia | 1 | Cardiotoxicity | 1 |
| Arrhythmia | 1 | Cerebellar ischaemia | 1 |
| Arterial Disorder | 1 | Cerebral vasoconstriction | 1 |
| Arthralgia | 1 | Circulatory collapse | 1 |
| Asthma | 1 | Condition aggravated | 1 |
| Back Pain | 1 | Confusional state | 1 |
| Blindness | 1 | Coronary artery disease | 1 |
| Blood Glucose Abnormal | 1 | Coronary artery stenosis | 1 |
| Blood Pressure Decreased | 1 | Cough | 1 |
| Blood Pressure Fluctuation | 1 | Device delivery system issue | 1 |
| Blood Thyroid Stimulating Hormone Increased | 1 | Device ineffective | 1 |
| Brain Fog | 1 | Device leakage | 1 |
| Bronchitis Chronic | 1 | Device occlusion | 1 |
| Brugada Syndrome | 1 | Device use issue | 1 |
| Cardiac Failure | 1 | Disorientation | 1 |
| Cardiac Murmur | 1 | Drug abuse | 1 |
| Cardiopulmonary Failure | 1 | Drug dose omission by device | 1 |
| Cardio-Respiratory Arrest | 1 | Drug resistance | 1 |
| Cerebrovascular Accident | 1 | Dysarthria | 1 |
| Chest Crushing | 1 | Electrocardiogram change | 1 |
| Circumoral Oedema | 1 | Electrocardiogram QRS complex abnormal | 1 |
| Circumstance Or Information Capable Of Leading To Medication Error | 1 | Electrocardiogram QT prolonged | 1 |
| Confusional State | 1 | Electrocardiogram ST-T change | 1 |
| Coronary Artery Stenosis | 1 | Electrocardiogram T wave inversion | 1 |
| Cough | 1 | Eye movement disorder | 1 |
| Cystitis Interstitial | 1 | Fatigue | 1 |
| Delirium | 1 | Feeling abnormal | 1 |
| Depression | 1 | Feeling hot | 1 |
| Device Use Issue | 1 | Flushing | 1 |
| Diarrhoea | 1 | Haemoptysis | 1 |
| Diastolic Dysfunction | 1 | Heart rate irregular | 1 |
| Dilatation Ventricular | 1 | Hemianopia | 1 |
| Drug Hypersensitivity | 1 | Hemiparesis | 1 |
| Dyspepsia | 1 | Hypersensitivity | 1 |
| Dysphonia | 1 | Hypoaesthesia | 1 |
| Dysstasia | 1 | Hypoxia | 1 |
| Ecg Signs Of Myocardial Ischaemia | 1 | Iatrogenic injury | 1 |
| Electrocardiogram Abnormal | 1 | Incorrect dosage administered | 1 |
| Face Oedema | 1 | Injection site haematoma | 1 |
| Feeling Jittery | 1 | Injection site ischaemia | 1 |
| Gastrointestinal Disorder | 1 | Injury associated with device | 1 |
| Haematemesis | 1 | Intentional product misuse | 1 |
| Haemodynamic Instability | 1 | Intracranial pressure increased | 1 |
| Haemorrhage | 1 | Irritability | 1 |
| Haemorrhage Intracranial | 1 | Ischaemia | 1 |
| Hallucination | 1 | Laryngeal oedema | 1 |
| Hemiplegia | 1 | Loss of consciousness | 1 |
| Hyperdynamic Left Ventricle | 1 | Memory impairment | 1 |
| Hypersomnia | 1 | Myocardial necrosis marker increased | 1 |
| Hypertensive Crisis | 1 | Neck pain | 1 |
| Ill-Defined Disorder | 1 | Nervous system disorder | 1 |
| Incoherent | 1 | Oedema | 1 |
| Injection Site Bruising | 1 | Off label use | 1 |
| Injection Site Discomfort | 1 | Oxygen saturation decreased | 1 |
| Injection Site Injury | 1 | Periorbital swelling | 1 |
| Injection Site Pruritus | 1 | Peripheral coldness | 1 |
| Intentional Product Misuse | 1 | Product design confusion | 1 |
| Intracranial Pressure Increased | 1 | Product quality issue | 1 |
| Memory Impairment | 1 | Pruritus | 1 |
| Migraine | 1 | Pulse absent | 1 |
| Muscle Spasms | 1 | Respiratory arrest | 1 |
| Muscular Weakness | 1 | Respiratory tract congestion | 1 |
| Musculoskeletal Stiffness | 1 | Restlessness | 1 |
| Myocardial Necrosis Marker Increased | 1 | Secretion discharge | 1 |
| Nasal Congestion | 1 | Supraventricular tachycardia | 1 |
| Nervousness | 1 | Syncope | 1 |
| New Daily Persistent Headache | 1 | Tetany | 1 |
| No Device Malfunction | 1 | Therapeutic product ineffective | 1 |
| Occupational Exposure To Product | 1 | Throat tightness | 1 |
| Oropharyngeal Oedema | 1 | Thrombosis in device | 1 |
| Pain In Extremity | 1 | Tongue oedema | 1 |
| Paraesthesia Oral | 1 | Toxicity to various agents | 1 |
| Periorbital Oedema | 1 | Troponin I increased | 1 |
| Peripheral Swelling | 1 | Troponin T increased | 1 |
| Pharyngeal Swelling | 1 | Ventricular dysfunction | 1 |
| Post-Traumatic Stress Disorder | 1 | Ventricular extrasystoles | 1 |
| Prescribed Overdose | 1 | Vision blurred | 1 |
| Presyncope | 1 | Wheezing | 1 |
| Product Administered At Inappropriate Site | 1 | Wrong dose | 1 |
| Product Preparation Issue | 1 | Wrong technique in product usage process | 1 |
| Productive Cough | 1 |  |  |
| Pulmonary Arterial Hypertension | 1 |  |  |
| Pulmonary Mass | 1 |  |  |
| Pulse Absent | 1 |  |  |
| Quadriplegia | 1 |  |  |
| Rash | 1 |  |  |
| Renal Failure | 1 |  |  |
| Respiratory Failure | 1 |  |  |
| Sinus Tachycardia | 1 |  |  |
| Sinusitis | 1 |  |  |
| Skin Discolouration | 1 |  |  |
| Somnolence | 1 |  |  |
| Stress | 1 |  |  |
| Stridor | 1 |  |  |
| Subarachnoid Haemorrhage | 1 |  |  |
| Supraventricular Tachycardia | 1 |  |  |
| Syncope | 1 |  |  |
| Tachypnoea | 1 |  |  |
| Therapy Non-Responder | 1 |  |  |
| Throat Tightness | 1 |  |  |
| Thunderclap Headache | 1 |  |  |
| Tinnitus | 1 |  |  |
| Tremor | 1 |  |  |
| Unmasking Of Previously Unidentified Disease | 1 |  |  |
| Vascular Stent Thrombosis | 1 |  |  |
| Vision Blurred | 1 |  |  |
| Weight Decreased | 1 |  |  |
| White Blood Cell Count Increased | 1 |  |  |
| **Related** | **N** | **Related** | **N** |
| Stress Cardiomyopathy | 16 | Tachycardia | 17 |
| Incorrect Route Of Product Administration | 13 | Stress cardiomyopathy | 16 |
| Heart Rate Increased | 11 | Headache | 10 |
| Hypotension | 10 | Incorrect route of product administration | 8 |
| Anaphylactic Reaction | 9 | Arteriospasm coronary | 7 |
| Headache | 9 | Chest pain | 6 |
| Myocardial Ischaemia | 9 | Hypertension | 6 |
| Acute Myocardial Infarction | 8 | Anaphylactic reaction | 5 |
| Dizziness | 8 | Anaphylactic shock | 5 |
| Overdose | 8 | Dyspnoea | 5 |
| Accidental Overdose | 7 | Incorrect dose administered | 5 |
| Blood Pressure Increased | 6 | Malaise | 5 |
| Cardiac Arrest | 6 | Accidental overdose | 4 |
| Cardiogenic Shock | 6 | Acute coronary syndrome | 4 |
| Chest Pain | 6 | Cardiac arrest | 4 |
| Hypertension | 6 | Hyperhidrosis | 4 |
| Accidental Exposure To Product | 5 | Hypotension | 4 |
| Arteriospasm Coronary | 5 | Pallor | 4 |
| Cardiomyopathy | 5 | Tremor | 4 |
| Drug Ineffective | 5 | Troponin increased | 4 |
| Injection Site Haemorrhage | 5 | Ventricular tachycardia | 4 |
| Loss Of Consciousness | 5 | Accidental exposure to product | 3 |
| Pulmonary Oedema | 5 | Acute myocardial infarction | 3 |
| Tachycardia | 5 | Blood pressure increased | 3 |
| Electrocardiogram St Segment Elevation | 4 | Cardiovascular disorder | 3 |
| Kounis Syndrome | 4 | Device failure | 3 |
| Off Label Use | 4 | Drug ineffective | 3 |
| Palpitations | 4 | Ischaemic stroke | 3 |
| Abdominal Pain | 3 | Medication error | 3 |
| Anxiety | 3 | Myocardial ischaemia | 3 |
| Cardiac Failure Acute | 3 | Palpitations | 3 |
| Hypokalaemia | 3 | Vasoconstriction | 3 |
| Incorrect Dose Administered | 3 | Ventricular fibrillation | 3 |
| Myocardial Infarction | 3 | Agitation | 2 |
| Nausea | 3 | Cardiogenic shock | 2 |
| Atrial Fibrillation | 2 | Cerebral haemorrhage | 2 |
| Bradycardia | 2 | Device malfunction | 2 |
| Brain Injury | 2 | Dizziness | 2 |
| Cardiac Disorder | 2 | Electrocardiogram repolarisation abnormality | 2 |
| Chest Discomfort | 2 | Electrocardiogram ST segment elevation | 2 |
| Condition Aggravated | 2 | Expired product administered | 2 |
| Device Failure | 2 | Extrasystoles | 2 |
| Dyspnoea | 2 | Heart rate increased | 2 |
| Electrocardiogram Qt Prolonged | 2 | Kounis syndrome | 2 |
| Expired Product Administered | 2 | Nausea | 2 |
| Feeling Abnormal | 2 | Product administration error | 2 |
| Heart Rate Decreased | 2 | Product dose omission issue | 2 |
| Hyperhidrosis | 2 | Product prescribing error | 2 |
| Hypersensitivity | 2 | Sinus tachycardia | 2 |
| Left Ventricular Dysfunction | 2 | Subarachnoid haemorrhage | 2 |
| Long Qt Syndrome | 2 | Ventricular arrhythmia | 2 |
| Malaise | 2 | Vomiting | 2 |
| Medication Error | 2 | Accidental exposure to product by child | 1 |
| Myocardial Stunning | 2 | Arrhythmia induced cardiomyopathy | 1 |
| Needle Issue | 2 | Arterial occlusive disease | 1 |
| Pallor | 2 | Artery dissection | 1 |
| Paraesthesia | 2 | Ataxia | 1 |
| Parosmia | 2 | Atrial fibrillation | 1 |
| Poor Peripheral Circulation | 2 | Bradycardia | 1 |
| Respiratory Distress | 2 | Burning sensation | 1 |
| Reversible Cerebral Vasoconstriction Syndrome | 2 | Cardiac failure | 1 |
| Shock | 2 | Cardiac fibrillation | 1 |
| Toxicity To Various Agents | 2 | Cardiotoxicity | 1 |
| Troponin T Increased | 2 | Cerebellar ischaemia | 1 |
| Ventricular Arrhythmia | 2 | Cerebral vasoconstriction | 1 |
| Ventricular Tachycardia | 2 | Chest discomfort | 1 |
| Acute Coronary Syndrome | 1 | Cold sweat | 1 |
| Acute Kidney Injury | 1 | Condition aggravated | 1 |
| Acute Pulmonary Oedema | 1 | Coronary artery disease | 1 |
| Acute Respiratory Distress Syndrome | 1 | Cyanosis | 1 |
| Alopecia | 1 | Device delivery system issue | 1 |
| Arrhythmia | 1 | Device leakage | 1 |
| Blood Glucose Abnormal | 1 | Device use issue | 1 |
| Blood Pressure Decreased | 1 | Drug abuse | 1 |
| Blood Pressure Fluctuation | 1 | Drug dose omission by device | 1 |
| Blood Thyroid Stimulating Hormone Increased | 1 | Drug hypersensitivity | 1 |
| Brain Fog | 1 | Drug resistance | 1 |
| Brugada Syndrome | 1 | Electrocardiogram QRS complex abnormal | 1 |
| Cardiopulmonary Failure | 1 | Electrocardiogram QT prolonged | 1 |
| Cerebral Haemorrhage | 1 | Electrocardiogram ST segment depression | 1 |
| Chest Crushing | 1 | Electrocardiogram ST-T change | 1 |
| Circumstance Or Information Capable Of Leading To Medication Error | 1 | Electrocardiogram T wave inversion | 1 |
| Confusional State | 1 | Eye movement disorder | 1 |
| Coronary Artery Stenosis | 1 | Feeling abnormal | 1 |
| Coronary Artery Thrombosis | 1 | Feeling hot | 1 |
| Cyanosis | 1 | Flushing | 1 |
| Depression | 1 | Hemianopia | 1 |
| Device Malfunction | 1 | Hemiparesis | 1 |
| Device Use Issue | 1 | Hypoaesthesia | 1 |
| Diastolic Dysfunction | 1 | Hypoxia | 1 |
| Dilatation Ventricular | 1 | Iatrogenic injury | 1 |
| Disseminated Intravascular Coagulation | 1 | Incorrect dosage administered | 1 |
| Drug Interaction | 1 | Injection site haematoma | 1 |
| Dyspepsia | 1 | Injection site ischaemia | 1 |
| Dysstasia | 1 | Injection site pain | 1 |
| Ecg Signs Of Myocardial Ischaemia | 1 | Injury associated with device | 1 |
| Electrocardiogram Abnormal | 1 | Intentional product misuse | 1 |
| Feeling Jittery | 1 | Intracranial pressure increased | 1 |
| Flushing | 1 | Irritability | 1 |
| Gastrointestinal Disorder | 1 | Ischaemia | 1 |
| Haematemesis | 1 | Left ventricular dysfunction | 1 |
| Hypersomnia | 1 | Loss of consciousness | 1 |
| Hypertensive Crisis | 1 | Myocardial infarction | 1 |
| Hypoxia | 1 | Myocardial necrosis marker increased | 1 |
| Injection Site Bruising | 1 | Myocardial oedema | 1 |
| Injection Site Discomfort | 1 | Oedema | 1 |
| Lactic Acidosis | 1 | Overdose | 1 |
| Migraine | 1 | Peripheral coldness | 1 |
| Muscular Weakness | 1 | Peripheral ischaemia | 1 |
| Myocardial Injury | 1 | Product quality issue | 1 |
| Myocardial Necrosis Marker Increased | 1 | Pruritus | 1 |
| Nervousness | 1 | Pulse absent | 1 |
| New Daily Persistent Headache | 1 | Rash | 1 |
| No Device Malfunction | 1 | Somnolence | 1 |
| Occupational Exposure To Product | 1 | Tetany | 1 |
| Paraesthesia Oral | 1 | Therapeutic product ineffective | 1 |
| Pharyngeal Swelling | 1 | Thrombosis in device | 1 |
| Post-Traumatic Stress Disorder | 1 | Toxicity to various agents | 1 |
| Prescribed Overdose | 1 | Troponin T increased | 1 |
| Presyncope | 1 | Ventricular dysfunction | 1 |
| Product Administered At Inappropriate Site | 1 | Ventricular hypokinesia | 1 |
| Product Preparation Issue | 1 | Vertigo | 1 |
| Pruritus | 1 | Vision blurred | 1 |
| Renal Failure | 1 | Wheezing | 1 |
| Sinus Tachycardia | 1 | Wrong dose | 1 |
| Skin Discolouration | 1 | Wrong technique in product usage process | 1 |
| Stress | 1 |  |  |
| Syncope | 1 |  |  |
| Therapy Non-Responder | 1 |  |  |
| Thunderclap Headache | 1 |  |  |
| Tinnitus | 1 |  |  |
| Tremor | 1 |  |  |
| Troponin Increased | 1 |  |  |
| Unmasking Of Previously Unidentified Disease | 1 |  |  |
| Unresponsive To Stimuli | 1 |  |  |
| Urticaria | 1 |  |  |
| Vasospasm | 1 |  |  |
| Ventricular Extrasystoles | 1 |  |  |
| Ventricular Hypokinesia | 1 |  |  |
| Vision Blurred | 1 |  |  |
| Weight Decreased | 1 |  |  |
| White Blood Cell Count Increased | 1 |  |  |
| **May be related** | **N** | **May be related** | **N** |
| Anaphylactic Reaction | 5 | Stress cardiomyopathy | 7 |
| Drug Ineffective | 3 | Bradycardia | 5 |
| Chest Discomfort | 2 | Drug ineffective | 5 |
| Condition Aggravated | 2 | Dyspnoea | 5 |
| Hypotension | 2 | Anaphylactic reaction | 4 |
| Nausea | 2 | Chest pain | 4 |
| Tachycardia | 2 | Ventricular tachycardia | 4 |
| Vomiting | 2 | Hypotension | 3 |
| Acute Coronary Syndrome | 1 | Myocardial infarction | 3 |
| Acute Myocardial Infarction | 1 | Palpitations | 3 |
| Agitation | 1 | Anaphylactic shock | 2 |
| Anaphylactic Shock | 1 | Blood pressure decreased | 2 |
| Arteriospasm Coronary | 1 | Cardiogenic shock | 2 |
| Blindness | 1 | Chest discomfort | 2 |
| Bradycardia | 1 | Kounis syndrome | 2 |
| Cardiac Arrest | 1 | Myocardial ischaemia | 2 |
| Cardiac Failure | 1 | Pallor | 2 |
| Cardiomyopathy | 1 | Sinus bradycardia | 2 |
| Chest Pain | 1 | Tachycardia | 2 |
| Delirium | 1 | Accidental overdose | 1 |
| Diarrhoea | 1 | Acute myocardial infarction | 1 |
| Electrocardiogram St Segment Elevation | 1 | Acute respiratory failure | 1 |
| Erythema | 1 | Altered state of consciousness | 1 |
| Face Oedema | 1 | Amnesia | 1 |
| Haemorrhage | 1 | Angina pectoris | 1 |
| Hallucination | 1 | Arteriospasm coronary | 1 |
| Headache | 1 | Arthralgia | 1 |
| Hemiplegia | 1 | Atrioventricular block | 1 |
| Hypertension | 1 | Autonomic nervous system imbalance | 1 |
| Hypoxia | 1 | Cardiac arrest | 1 |
| Incoherent | 1 | Cardiac failure | 1 |
| Incorrect Route Of Product Administration | 1 | Circulatory collapse | 1 |
| Injection Site Pruritus | 1 | Cold sweat | 1 |
| Intentional Product Misuse | 1 | Coronary artery stenosis | 1 |
| Kounis Syndrome | 1 | Cough | 1 |
| Lactic Acidosis | 1 | Cyanosis | 1 |
| Loss Of Consciousness | 1 | Drug hypersensitivity | 1 |
| Muscle Spasms | 1 | Ejection fraction decreased | 1 |
| Musculoskeletal Stiffness | 1 | Electrocardiogram change | 1 |
| Myocardial Injury | 1 | Electrocardiogram ST segment depression | 1 |
| Myocardial Ischaemia | 1 | Electrocardiogram ST segment elevation | 1 |
| Myocardial Stunning | 1 | Fatigue | 1 |
| Off Label Use | 1 | Haemoptysis | 1 |
| Pruritus | 1 | Headache | 1 |
| Quadriplegia | 1 | Heart rate increased | 1 |
| Rash | 1 | Heart rate irregular | 1 |
| Sinusitis | 1 | Hyperhidrosis | 1 |
| Stress Cardiomyopathy | 1 | Incorrect dose administered | 1 |
| Stridor | 1 | Incorrect route of product administration | 1 |
| Supraventricular Tachycardia | 1 | Injection site pain | 1 |
| Toxicity To Various Agents | 1 | Left ventricular dysfunction | 1 |
| Troponin Increased | 1 | Memory impairment | 1 |
| Unresponsive To Stimuli | 1 | Neck pain | 1 |
| Urticaria | 1 | Nervous system disorder | 1 |
| Vasospasm | 1 | Oxygen saturation decreased | 1 |
| Ventricular Extrasystoles | 1 | Periorbital swelling | 1 |
| Ventricular Hypokinesia | 1 | Product use complaint | 1 |
|  |  | Pulmonary oedema | 1 |
|  |  | Respiratory arrest | 1 |
|  |  | Respiratory tract congestion | 1 |
|  |  | Secretion discharge | 1 |
|  |  | Somnolence | 1 |
|  |  | Throat tightness | 1 |
|  |  | Tongue oedema | 1 |
|  |  | Urticaria | 1 |
|  |  | Ventricular extrasystoles | 1 |
|  |  | Ventricular fibrillation | 1 |
|  |  | Ventricular hypokinesia | 1 |
|  |  | Vomiting | 1 |
| **May have contributed** | **N** | **May have contributed** | **N** |
| Anaphylactic Reaction | 9 | Acute myocardial infarction | 4 |
| Drug Ineffective | 9 | Myocardial ischaemia | 4 |
| Hypotension | 9 | Cardiac arrest | 3 |
| Tachycardia | 5 | Chest pain | 3 |
| Myocardial Ischaemia | 4 | Tachycardia | 3 |
| Cardiac Arrest | 3 | Anaphylactic reaction | 2 |
| Dyspnoea | 2 | Atrial fibrillation | 2 |
| Hypertension | 2 | Incorrect route of product administration | 2 |
| Hypoxia | 2 | Amnesia | 1 |
| Kounis Syndrome | 2 | Anaphylactic shock | 1 |
| Nausea | 2 | Angina pectoris | 1 |
| Wheezing | 2 | Arterial spasm | 1 |
| Accidental Overdose | 1 | Cardiac failure congestive | 1 |
| Acquired Left Ventricle Outflow Tract Obstruction | 1 | Cardiomyopathy | 1 |
| Acute Myocardial Infarction | 1 | Chest discomfort | 1 |
| Anaphylactic Shock | 1 | Confusional state | 1 |
| Arterial Disorder | 1 | Device ineffective | 1 |
| Arthralgia | 1 | Device occlusion | 1 |
| Asthma | 1 | Disorientation | 1 |
| Back Pain | 1 | Drug hypersensitivity | 1 |
| Blood Pressure Increased | 1 | Drug ineffective | 1 |
| Bradycardia | 1 | Dysarthria | 1 |
| Brain Injury | 1 | Dyspnoea | 1 |
| Bronchitis Chronic | 1 | Ejection fraction decreased | 1 |
| Cardiac Murmur | 1 | Electrocardiogram ST segment elevation | 1 |
| Cardio-Respiratory Arrest | 1 | Headache | 1 |
| Cerebral Haemorrhage | 1 | Heart rate increased | 1 |
| Cerebrovascular Accident | 1 | Hypersensitivity | 1 |
| Chest Discomfort | 1 | Hypertension | 1 |
| Circumoral Oedema | 1 | Hypotension | 1 |
| Condition Aggravated | 1 | Kounis syndrome | 1 |
| Coronary Artery Thrombosis | 1 | Laryngeal oedema | 1 |
| Cough | 1 | Myocardial infarction | 1 |
| Cyanosis | 1 | Myocardial oedema | 1 |
| Cystitis Interstitial | 1 | Nausea | 1 |
| Device Malfunction | 1 | Off label use | 1 |
| Disseminated Intravascular Coagulation | 1 | Overdose | 1 |
| Dizziness | 1 | Palpitations | 1 |
| Drug Hypersensitivity | 1 | Peripheral ischaemia | 1 |
| Drug Interaction | 1 | Product design confusion | 1 |
| Dysphonia | 1 | Product use complaint | 1 |
| Erythema | 1 | Pulmonary oedema | 1 |
| Expired Product Administered | 1 | Rash | 1 |
| Flushing | 1 | Restlessness | 1 |
| Haemodynamic Instability | 1 | Sinus tachycardia | 1 |
| Haemorrhage Intracranial | 1 | Stress cardiomyopathy | 1 |
| Headache | 1 | Supraventricular tachycardia | 1 |
| Hyperdynamic Left Ventricle | 1 | Syncope | 1 |
| Hyperhidrosis | 1 | Tremor | 1 |
| Hypersensitivity | 1 | Troponin I increased | 1 |
| Ill-Defined Disorder | 1 | Troponin increased | 1 |
| Incorrect Dose Administered | 1 | Urticaria | 1 |
| Incorrect Route Of Product Administration | 1 | Ventricular fibrillation | 1 |
| Injection Site Injury | 1 | Vertigo | 1 |
| Intracranial Pressure Increased | 1 |  |  |
| Lactic Acidosis | 1 |  |  |
| Memory Impairment | 1 |  |  |
| Nasal Congestion | 1 |  |  |
| Oropharyngeal Oedema | 1 |  |  |
| Pain In Extremity | 1 |  |  |
| Periorbital Oedema | 1 |  |  |
| Peripheral Swelling | 1 |  |  |
| Productive Cough | 1 |  |  |
| Pruritus | 1 |  |  |
| Pulmonary Arterial Hypertension | 1 |  |  |
| Pulmonary Mass | 1 |  |  |
| Pulse Absent | 1 |  |  |
| Respiratory Failure | 1 |  |  |
| Reversible Cerebral Vasoconstriction Syndrome | 1 |  |  |
| Somnolence | 1 |  |  |
| Subarachnoid Haemorrhage | 1 |  |  |
| Tachypnoea | 1 |  |  |
| Throat Tightness | 1 |  |  |
| Troponin T Increased | 1 |  |  |
| Urticaria | 1 |  |  |
| Vascular Stent Thrombosis | 1 |  |  |
| Ventricular Arrhythmia | 1 |  |  |
| Ventricular Extrasystoles | 1 |  |  |
| Vomiting | 1 |  |  |
